# Studies on the PII-PipX-NtcA Regulatory Axis of Cyanobacteria Provide Novel Insights into the Advantages and Limitations of Two-Hybrid Systems for Protein Interactions

**DOI:** 10.3390/ijms25105429

**Published:** 2024-05-16

**Authors:** Paloma Salinas, Sirine Bibak, Raquel Cantos, Lorena Tremiño, Carmen Jerez, Trinidad Mata-Balaguer, Asunción Contreras

**Affiliations:** Departamento. de Fisiología, Genética y Microbiología, Universidad de Alicante, 03690 San Vicente del Raspeig, Spain; paloma.salinas@ua.es (P.S.); sb221@alu.ua.es (S.B.); raquel.cantos@ua.es (R.C.); lorena.tremino@ua.es (L.T.); carmen.jerez@ua.es (C.J.); trinidad.mata@ua.es (T.M.-B.)

**Keywords:** protein-protein interaction, nitrogen interaction network, *Synechococcus elongatus* PCC7942, yeast two-hybrid, bacterial two-hybrid

## Abstract

Yeast two-hybrid approaches, which are based on fusion proteins that must co-localise to the nucleus to reconstitute the transcriptional activity of GAL4, have greatly contributed to our understanding of the nitrogen interaction network of cyanobacteria, the main hubs of which are the trimeric PII and the monomeric PipX regulators. The bacterial two-hybrid system, based on the reconstitution in the *E. coli* cytoplasm of the adenylate cyclase of *Bordetella pertussis*, should provide a relatively faster and presumably more physiological assay for cyanobacterial proteins than the yeast system. Here, we used the bacterial two-hybrid system to gain additional insights into the cyanobacterial PipX interaction network while simultaneously assessing the advantages and limitations of the two most popular two-hybrid systems. A comprehensive mutational analysis of PipX and bacterial two-hybrid assays were performed to compare the outcomes between yeast and bacterial systems. We detected interactions that were previously recorded in the yeast two-hybrid system as negative, as well as a “false positive”, the self-interaction of PipX, which is rather an indirect interaction that is dependent on PII homologues from the *E. coli* host, a result confirmed by Western blot analysis with relevant PipX variants. This is, to our knowledge, the first report of the molecular basis of a false positive in the bacterial two-hybrid system.

## 1. Introduction

Cyanobacteria, phototrophic prokaryotes that perform oxygenic photosynthesis, are the main contributors to marine primary production [1] and have a very important ecological impact on global carbon, nitrogen and oxygen cycles. They have evolved sophisticated systems to maintain the homeostasis of carbon/nitrogen assimilation (reviewed in [2,3]), the two most abundant elements in all living forms. Cyanobacteria can use different nitrogen sources that are first converted into ammonium and then incorporated into amino acids and other N-containing compounds via the glutamine synthetase-glutamate synthase (GS-GOGAT) pathway, using 2-oxoglutarate (2-OG) as a carbon skeleton [4,5,6].

In bacteria and plants, 2-OG, a universal indicator of the intracellular carbon-to-nitrogen balance [7,8], is sensed by the widely distributed and highly conserved signal transduction protein PII [9,10]. Homotrimeric PII proteins are encoded by two paralog genes (*glnB* and *glnK*) in enterobacteria and by just one representative (*glnB*) in cyanobacteria and plants. PII regulates the activity of proteins involved in nitrogen and carbon metabolism by direct protein–protein interactions, perceiving metabolic information through the competitive binding of ATP or ADP and the synergistic binding of ATP and 2-OG [11,12,13,14]. Despite the remarkable structural conservation of PII proteins across phyla, unique PII targets are found in cyanobacteria.

The yeast two-hybrid system (Y2H), based on fusion proteins that must co-localise to the yeast nucleus to reconstitute the transcriptional activity of GAL4 [15], has been shown to closely reflect the functional interactions mediated by enterobacterial [16,17,18] or cyanobacterial [19,20,21,22,23,24,25] nitrogen regulators. The first PII receptors identified in cyanobacteria were detected in a Y2H search for proteins of the unicellular strain *Synechococcus elongatus* PCC7942 (hereafter *S. elongatus*) interacting with PII: N-acetyl glutamate kinase (NAGK), which form complexes with PII in cyanobacteria and plants [26,27] and PipX (PII interacting protein X), a small protein of 89 amino acids that is restricted to cyanobacteria [19,21,28,29]. PipX is composed of an N-terminal TLD/KOW domain [30] and a C-terminal domain of two alpha-helices, the first of which contains a basic arginine-rich patch with a function that remains enigmatic [31,32].

PipX was also found as prey in yeast two-hybrid searches with the global transcriptional regulator NtcA, which is involved in nitrogen assimilation in cyanobacteria [33,34,35,36]. PipX provides a mechanistic link between PII signalling and gene expression in response to nitrogen limitation [21,37]. The PipX–NtcA complex consists of one active (2-OG bound) NtcA dimer and two PipX molecules. Each NtcA subunit binds one PipX molecule. PipX stabilises the conformation of NtcA, which is transcriptionally active and probably helps the local recruitment of RNA polymerase. PipX uses the same surface of its TLD/KOW domain to bind to either 2-OG-bound NtcA, stimulating DNA binding and transcriptional activity, or to 2-OG-free PII. The PII sequestration of PipX at low 2-OG renders PipX unavailable for NtcA binding and activation, reducing the expression of NtcA-dependent gene targets [20,23,25,36,38,39]. In addition, the interaction between PII and PipX is highly sensitive to fluctuations in the ATP/ADP ratio and, thus, the energy state of the cells [40].

The bacterial two-hybrid system (BACTH), a genetic approach to protein–protein interaction (PPI) that is based on the reconstitution in *E. coli* of the adenylate cyclase of *Bordetella pertussis* from its T18 and T25 domains, fused to the proteins of interest [41,42], has been successfully used to identify components and unravel the molecular details of interaction networks involved in cell division or heterocyst patterning in cyanobacteria [43,44,45,46]. We recently used the BACTH system to prove interactions between PipX and the ribosome-assembly GTPase EngA, two proteins whose interactions in cyanobacteria were previously inferred by synteny [47] and in vivo [48] approaches. It is worth noting that while BACTH assays gave robust interaction signals between PipX and EngA [48], the Y2H system did not. However, false negatives are common in both types of two-hybrid systems and often depend on the pair of proteins assayed, although the reasons behind this fact are not always obvious. Since the binding of a given partner to PipX in *S. elongatus* depends on the levels of the effectors favouring the corresponding complexes, we wondered whether the BACTH system is a more appropriate choice than the Y2H to investigate cyanobacterial regulatory networks.

The aim of this work was to gain additional insights into the cyanobacterial nitrogen interaction network and the advantages and limitations of the most popular two-hybrid systems. PipX mutations that were previously analysed by Y2H assays or in other contexts were now analysed with the BACTH system for self-interactions or cross-interactions with NtcA or PII, its two best-known partners, and with GlnB or GlnK, the *E. coli* homologs of PII. Interactions reported as negative in the Y2H system, as well as indirect interactions dependent on host proteins, were detected, the results speaking in favour of the greater biological complexity of BACTH assays in the present context. Complementary Western blot assays with the relevant PipX variants supported the main inferences.

## 2. Results and Discussion

### 2.1. Interactions Involving PipX-PII and PipX-NtcA

**Figure 1:**
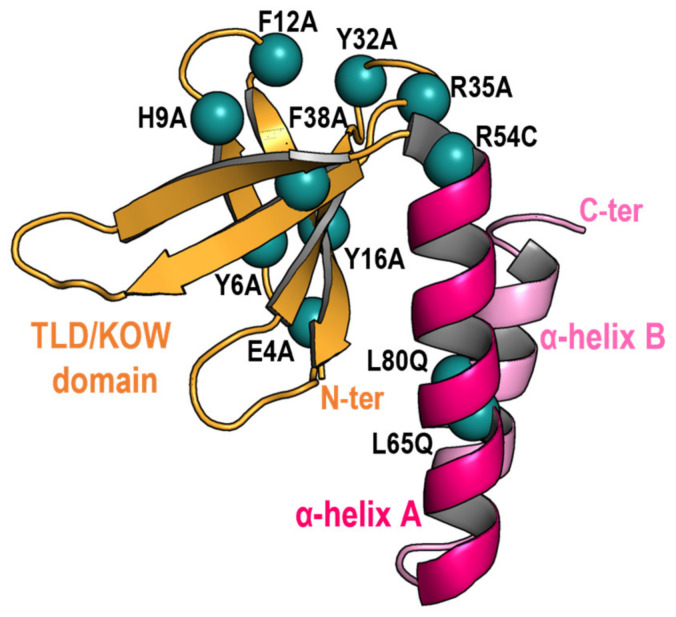
Location of residues (blue spheres) mutated on the PipX structure (chain E of the PDB file 2XG8), which is shown in cartoon representation with the N-terminal TLD/KOW domain and the C-terminal helices coloured in orange and pink, respectively.

Multiple factors can affect the results of two-hybrid assays, including the possible occlusion of interaction determinants by the domains added to the tested proteins, or, importantly, biological factors differing between the systems. Thus, comparing results from Y2H and BACTH systems may provide complementary information on promiscuous proteins such as PipX. For instance, we found in [48] that while PipX interactions with PII are almost as easy to detect in BACTH as in Y2H assays, the interactions between PipX and NtcA were detected better with the BACTH system [20,23,48], perhaps due to lower levels of 2-OG in the yeast nucleus. To gain additional insights into the PipX interaction network while comparing the outcomes from BACTH and Y2H assays, we performed BACTH assays with representative PipX point mutations that were previously analysed by Y2H [20,23,25,49]. The positions of the PipX residues discussed in this work are illustrated in Figure 1.

To carry out BACTH assays, we first introduced the point mutations of interest into PipX-T18 or T18-PipX constructs to generate the corresponding PipX*-T18 or T18-PipX* derivatives (Table 1 and Table S1 in the Supplementary Materials). PipX*-T18 derivatives were tested against T25-PII or T25-NtcA, while T18-PipX* derivatives were only tested against T25-NtcA, since the weak interaction signal produced by the control pair (T18-PipX/T25-PII [48]) makes this combination hardly informative [48]. To discriminate amongst the different levels of interaction signals with reasonable confidence on plate assays, three independent pools of five or six clones each, taken from two independent transformations, were tested in parallel on two different media for each assay. In this way, we obtained information from both *lac* and *mal* reporters. According to their colour (red or blue, depending on the indicator media), intensity signals were visually classified into five categories ranging from “no interaction” to “very strong”, exactly as described previously [48]. The “very strong” class applies to the maximal signals obtained so far between *S. elongatus* tested proteins. Note that while the strength of the signal is not a direct measure of protein affinity, ranking the different levels of signals from equivalent fusion proteins does allow the comparison of protein variants in the BACTH system, assuming that these are equally expressed.

**Figure 2:**
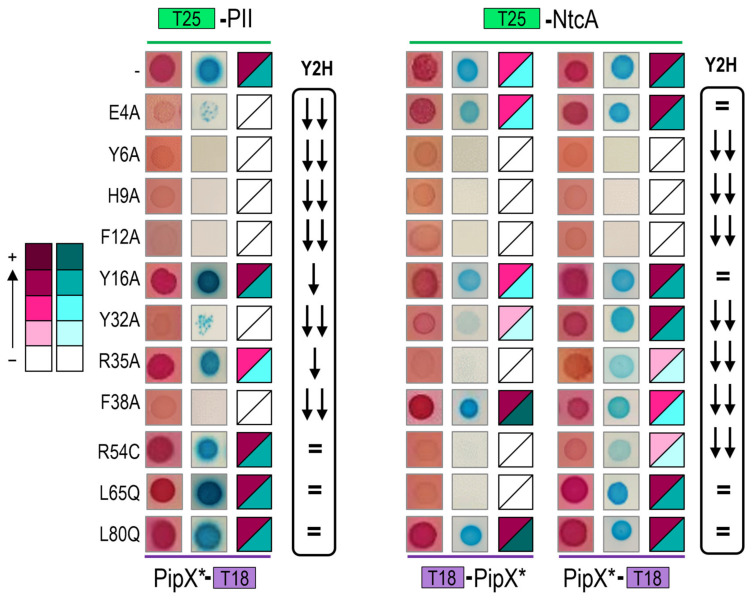
Effect of PipX point mutations (*) on two-hybrid interaction signals with PII and NtcA. The colour scale, from no interaction (−) to the highest (+) interaction signals, is shown on the left. In each case, representative photographs from a minimum of six assays and heatmaps summarizing the BACTH results on MacConkey-lactose or M63-maltose-X-gal are shown from left to right. The relative position of the T18 or T25 domains is illustrated in each case. The Y2H column indicates the impact of the corresponding mutations in reported Y2H assays: no effect, significant effect and very drastic effect are indicated by an equals sign, one, or two arrows, respectively.

The results of the BACTH analysis are illustrated in Figure 2, with representative photographs and heatmaps summarising the corresponding information for PipX*/PII and PipX*/NtcA pairs, respectively. For comparison, the impact of the same mutations on Y2H signals corresponding to the same pairs [20,23,25,49] is represented to the right. For simplicity, Y2H signals are classified into just three categories to denote the impact of mutations on the strength of the signal: no effect, significant effect, or very drastic effect.

Highly concordant results from the two types of assays were obtained for most mutations, particularly for PipX*/PII pairs. The concordance was very clear for the most drastic mutations, that is, those abolishing or significantly impairing signals with both partners (Y6A, H9A and F12A), or just with PII (E4A) or with NtcA (R54C), in Y2H assays. Concordance was also observed for two control mutations (Y16A and L80Q) that had no effects on the PipX-PII or PipX-NtcA interactions tested.

**Figure 3:**
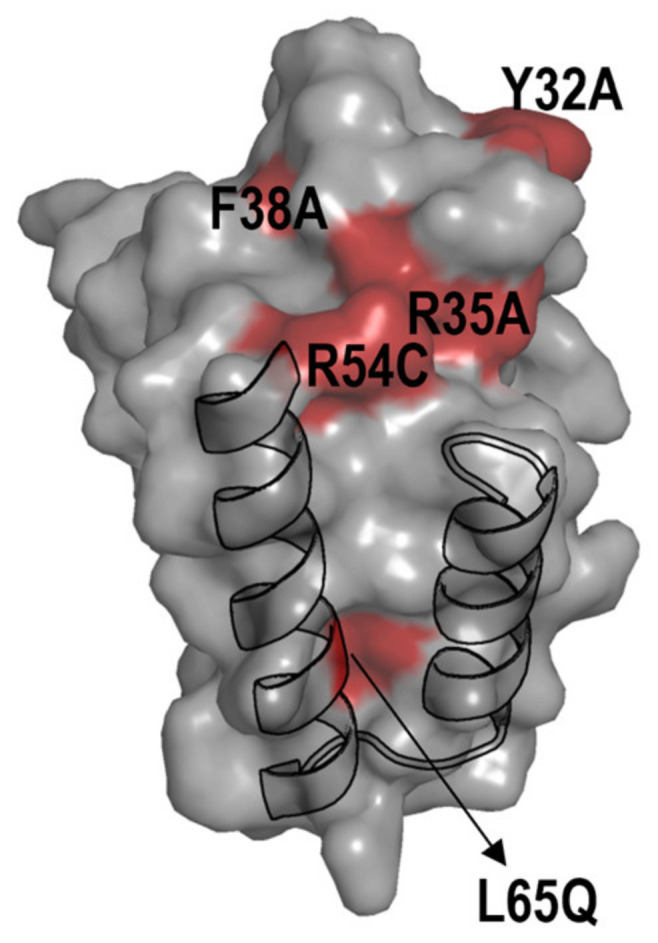
Location on the PipX surface of mutated residues for which the BACTH system was particularly informative. The surface structure of the PipX subunit (chain E of the PDB file 2XG8) is represented in semi-transparent form, rendering visible the flexed C-terminal helices in cartoon representation. Surface regions of PipX corresponding to mutated residues with discordant results are coloured in red.

The PipX*/NtcA comparisons resulted in discrepancies for several residues whose locations on the PipX surface are illustrated in Figure 3.

Relatively weak but significant BACTH signals with NtcA were produced by certain variants (Y32A, R35A, F38A and R54C) that gave no signals in the Y2H analysis [20,23,25], suggesting that the previous Y2H assays overestimated the impact of mutations on interactions with NtcA. Importantly, the results with those four PipX variants, showing their ability to interact with NtcA in the BACTH system, are easily reconciled with phenotypes observed in *S. elongatus*, where the mutations R35A, F38A and R54C did not abolish NtcA coactivation and the mutation Y32A increased it [20,23,37].

Conversely, L65Q, a mutation having no impact in the Y2H system, decreased BACTH interaction signals specifically with NtcA. Since L65 is outside the TLD/KOW domain providing the contacts with NtcA, the reason for the rather drastic effect of L65Q on interactions with NtcA in the BACTH system is unclear. However, L65Q was identified as a spontaneous mutation suppressing PipX toxicity and it has recently been shown to decrease PipX levels in *S. elongatus* [20,54], which suggests the reduced formation of in vivo complexes between PipX^L65Q^ and PII. Thus, interaction or functional assays indicated that the L65Q mutation did have phenotypic consequences that differed for PipX-PII and PipX-NtcA complexes. Our interpretation of the discrepancies between the different assays is that unknown bacterial factors, which are absent in Y2H assays, are differentially affecting PipX-PII and PipX-NtcA complexes in the corresponding in vivo assays. In this context, it is tempting to propose that PipX-NtcA complexes may be stabilised in *E. coli* by a relatively abundant factor and that the C-terminal helices are involved in that interaction. Based on recent structural data on complexes between NtcA and RNApol from *Anabaena* that do not include PipX [55], it is tempting to propose that an RNApol subunit may provide just such an interacting partner in *E. coli*.

In summary, the BACTH assays performed here in the context of PipX interactions with PII or NtcA support the usefulness of both PPI methods for mutational analyses, further expanding the inferences and conclusions derived from previous Y2H studies.

### 2.2. PipX Interacts with E. coli GlnB and GlnK Proteins in the BACTH System

The *E. coli* cytoplasm apparently provides a more biologically informative context than the yeast nucleus for cyanobacterial proteins, but it would also provide a comparatively more complex environment due to the higher abundance of more closely related proteins and, thus, of potential interacting partners of the protein(s) being assayed. Since those bacterial proteins may affect the BACTH interactions by either competing for binding or by acting as a bridge, they would respectively contribute to false negatives or false positives. It is worth noting that while, in the Y2H system, false positives are common due to the “stickiness” of the GAL4 domain, this does not happen in the BACTH system, where there are no reports of false positives [56,57]. Because of this, interactions giving positive results in the BACTH system must be considered informative and worth investigating, even if they cannot be confirmed by in vitro assays.

An intriguing “false positive” obtained by us in previous BACTH assays was the strong self-interaction of PipX [48], given that gel filtration assays [25] and a lack of self-interaction in Y2H analyses [21,23,25] indicated that PipX is monomeric. However, PipX self-interaction was observed in yeast three-hybrid (Y3H) assays when PII was used as a bridge protein [25] and we reasoned that the *E. coli* proteins GlnB and GlnK, by providing a bridge, could be responsible for the PipX self-interaction observed in *E. coli*. However, Y2H assays did not support such interactions between PipX and *E. coli* GlnB or GlnK [21] and, thus, the mechanism behind PipX-self interactions in the BACTH system remained enigmatic.

To investigate whether PipX-self interaction is facilitated by *E. coli* GlnB and/or GlnK proteins in BACTH assays, we first determined whether we could detect cross-interaction between PipX and GlnB and/or GlnK proteins in the BACTH system. To this end, we produced N-terminal fusions of each of the T18 and T25 domains to GlnB or GlnK, to test the interactions between PipX and GlnB and/or GlnK against the corresponding PipX derivatives. For comparison, *S. elongatus* PII fusions were also tested in parallel, giving a total of 12 pairs of fusion derivatives to be assayed for cross-interactions.

**Figure 4:**
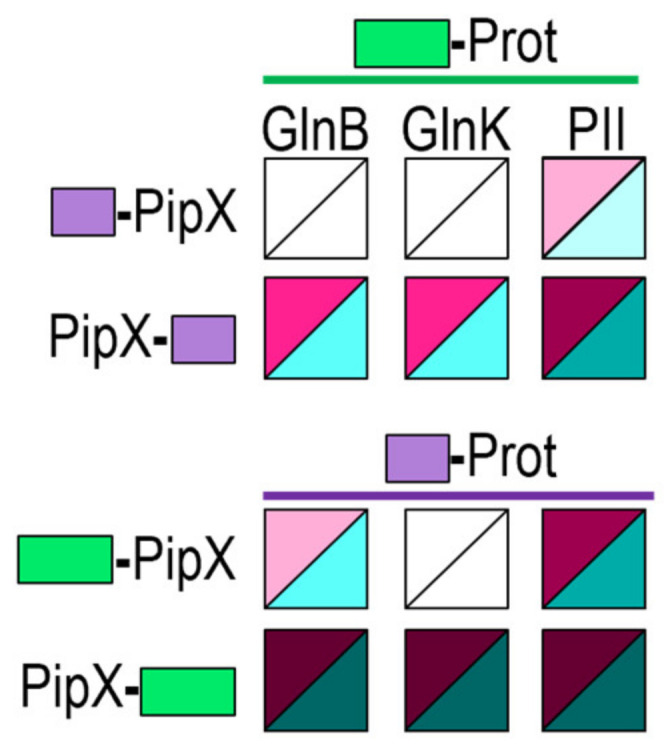
PipX interacts with GlnB and GlnK proteins in the BACTH system. Heatmaps summarise the results for the indicated fusion proteins. Additional data are provided in Figure S1 in the Supplementary Materials. Other details are as shown in Figure 2.

As shown in Figure 4 and Figure S1 in the Supplementary Materials, and in close agreement with previous results [48], PII/PipX pairs gave positive results in all combinations tested (4/4). In addition, and at odds with previous Y2H assays [21], positive results were also obtained for some of the GlnB/PipX (3/4) or GlnK/PipX (2/4) pairs, indicating that PipX has an affinity for each of these two *E. coli* proteins. Since the levels of the interaction signals for GlnB/PipX or GlnK/PipX positive pairs were always lower than those for PII/PipX pairs, altogether, the results indicate that the affinity of PipX for the *E. coli* proteins may be significantly lower than for *S. elongatus* PII.

It is worth noting that the intensity of the interaction signals and the levels to which protein fusions accumulate are mutually interdependent in the BACTH system, due to the positive role of cAMP in the expression of the fusion proteins [56]. This implies that initial differences of expression between fusion proteins will be magnified when interacting pairs reconstitute the adenylate cyclase activity. Therefore, differences in expression between the studied proteins due to differences in codon usage and/or stability between the yeast and *E. coli* hosts would contribute to discrepancies between the two systems. In this context, it appears reasonable that the expression of the particularly abundant proteins GlnB and GlnK would be highly optimised in *E. coli*, resulting in higher levels of BACTH fusion derivatives in comparison with PII derivatives, in turn overestimating the strength of the GlnB-PipX or GlnK-PipX interactions.

### 2.3. Self- and Cross-Interactions Involving PII, GlnB and GlnK Proteins, and the Differences between Two-Hybrid Systems

An additional complication for our BACTH interaction assays is the possibility of heterotrimerisation between the endogenous host proteins GlnB or GlnK and *S. elongatus* PII derivatives. Heterotrimerisation between *S. elongatus* PII and GlnB or GlnK, which was first suggested from in vivo studies [58], has been demonstrated by in vitro [59] and Y2H assays [19]. While it is expected that heterotrimerisation with the endogenous proteins [60] could interfere with the self-interactions from each of the three homologs in the BACTH system, the impact of interference would be greater for the less abundant fusion protein, which is likely to be *S. elongatus* PII.

**Figure 5:**
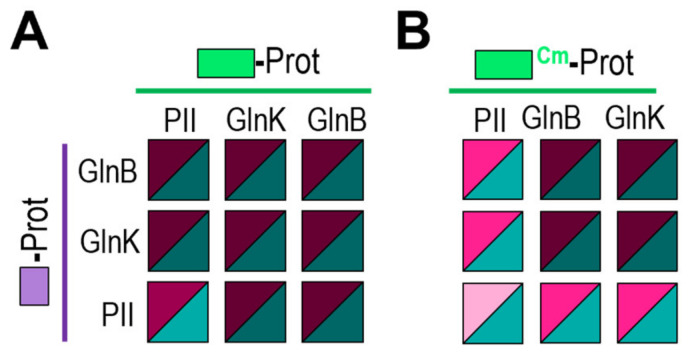
PII gives weaker self- and cross-interaction signals than GlnB or GlnK in the BACTH system. T25 derivatives were expressed from pKT25 vector (**A**) or pT25 vector (**B**). Additional data are provided in Figure S1 in the Supplementary Materials. Other details are as those in Figure 2.

To explore this issue, we first compared interactions involving the three proteins in all nine compatible pair combinations (Figure 5A and Figure S1 in the Supplementary Materials). As expected, all of them were positive, in line with their ability to form homo and heterotrimers. In addition, the weakest signals corresponded to PII self-interaction. This is at odds with previous Y2H assays that gave very strong self-interaction signals for PII, signals that were, in fact, stronger than the self-interaction signals for GlnB or GlnK and stronger than cross-interactions between PII and GlnB or GlnK [19,21]. These discrepancies are in complete agreement with the idea that BACTH interaction signals reflect comparatively lower levels of expression of *S. elongatus* PII derivatives, rather than differences in binding affinities, which are not expected to be lower for PII/PII than for PII/GlnB or PII/GlnK interacting pairs.

### 2.4. PII Gives Weaker Interaction Signals than GlnB and GlnK Proteins in the BACTH System

Detection of the relatively small differences between interacting pairs appears to be prevented by the saturation of the signal, presumably due to the very high levels of expression obtained for GlnB or GlnK derivatives. To test this idea, we next expressed T25-GlnB or T25-GlnK fusion proteins from an essentially identical BACTH vector from which fusion proteins are less efficiently expressed. For simplicity, we refer to the corresponding protein fusions as T25^Cm^-GlnB or T25^Cm^-GlnK, making reference to the selection marker (Cm instead of Km; see Section 3 and Table 1).

Still robust, but significantly weaker signals were obtained for PII/GlnB and PII/GlnK and were obtained to a greater extent for PII/PII pairs when T25^Cm^-PII, T25^Cm^-GlnB or T25 ^Cm^-GlnK were involved (Figure 5B and Figure S1 in the Supplementary Materials), further supporting our inference of *S. elongatus* PII derivatives being expressed at lower levels in the BACTH system. Conversely, the signals were still maximal for either self-interactions or cross-interactions mediated by GlnB and/or GlnK, a result in line with the comparatively higher levels of the *E. coli* fusion proteins in the system.

Differences in codon usage or susceptibility to *E. coli* proteases may contribute to the comparatively low levels of expression of *S. elongatus* PII, and presumably of the other cyanobacterial proteins analysed in this work. These results call attention to the contribution of expression levels/protein stability to the strength of the interaction signals in the BACTH system.

### 2.5. Mutations Impairing PipX Self-Interactions Also Impair Binding to GlnK or GlnB in E. coli

**Figure 6:**
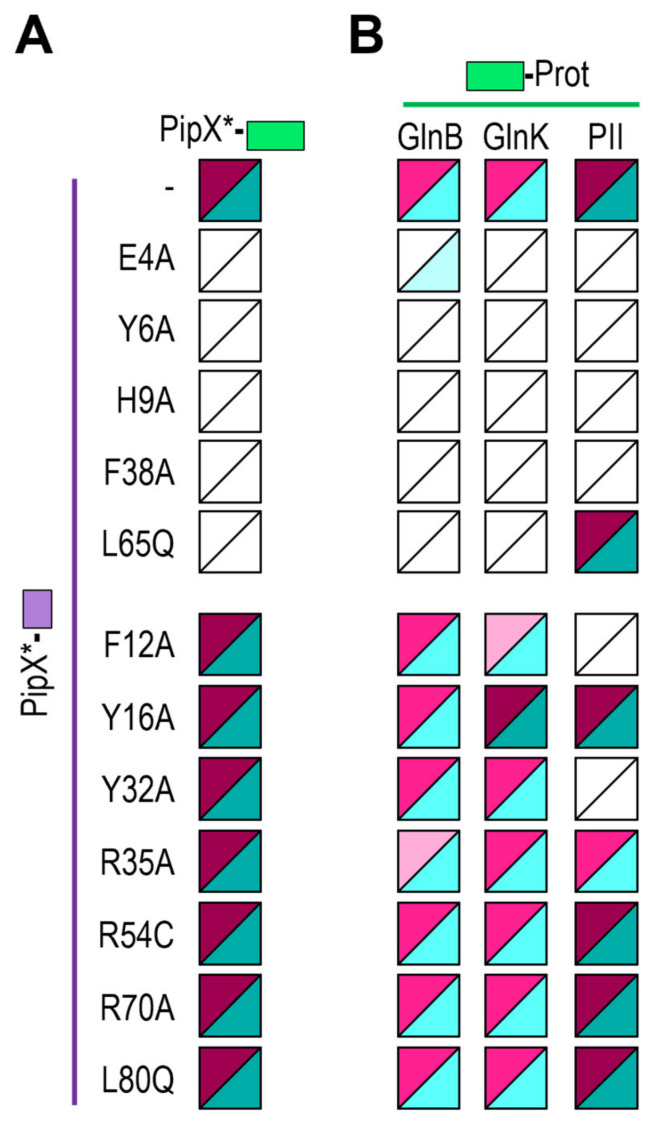
Effect of PipX point mutations (*) on self- and cross-interactions with GlnB or GlnK in the BACTH system. (**A**) Self-interactions; (**B**) cross-interactions. Additional data are provided in Figure S2 in the Supplementary Materials. Other details are as those in Figure 2.

BACTH analysis with deletions eliminating the C-terminal domain or just the last α-helix (helix B) of PipX indicated that both the TLD/KOW domain and the helix A are required for maximal self-interaction signals [48]. Since we hypothesised that self-interaction could be facilitated by PipX trimerisation over GlnB and/or GlnK in *E. coli*, it was important to determine whether the determinants for PipX self-interactions coincided with PipX determinants for binding to GlnB and/or GlnK. To this end, PipX*-T25 derivatives (Table 1 and Table S2 in the Supplementary Materials) were next tested against their cognate PipX*-T18 derivatives to determine the impact of each of the PipX point mutations on PipX self-interaction. The results of self-interaction assays for each of the mutant versions are summarised in Figure 6A and Figure S2 in the Supplementary Materials.

In total, 5 out of the 12 tested point mutations impaired interaction signals and all except one of the mutations impairing self-interaction (L65Q at helix A) also target the N-terminal domain, thus supporting the importance of the TLD/KOW domain and helix A of PipX for self-interaction.

Once we had identified those residues where the mutations are either neutral or impair PipX self-interaction in *E. coli*, we next determined whether these PipX mutations had similar effects on interactions with GlnB or GlnK. To this end, we tested the set of PipX*-T18 derivatives against T25-GlnB or T25-GlnK (Figure 6B and Figure S2 in the Supplementary Materials). To facilitate discussion and comparisons, we grouped separately those results corresponding to mutations impairing (top) or not impairing (bottom) self-interactions and included the heatmaps previously obtained with PipX*/PII at the right.

The mutations impairing PipX self-interactions could be classed into two interaction patterns: those impairing signals with all three PII, GlnB and GlnK proteins (E4A, Y6A, H9A, and F38A) and those impairing signals just with GlnB and GlnK (L65Q). Importantly, none of them were neutral or increased interaction signals with GlnB. Conversely, the mutations having no effect on self-interactions (F12A, Y16A, Y32A, R35A, R54C, R70A, and L80Q) did not abolish signals with either GlnB or GlnK. One of them (R35A) slightly decreased the interaction signals with GlnB, while another one (Y16A) slightly increased them with GlnK. Last, but not least, two of the neutral mutations in the context of PipX self-interactions (F12A, Y32A) impaired just the signals with PII.

Therefore, the results strongly support the hypothesis that PipX self-interaction in the BACTH assays is due to the presence of GlnB and/or GlnK proteins and call attention to the ability of the system to detect indirect interactions, particularly from abundant host proteins such as GlnB or GlnK.

### 2.6. Mutations Impairing PipX Binding to GlnK or GlnB in E. coli Also Impair PipX Levels in E. coli

Because PipX mutations impairing binding to PII also impair PipX levels in *S. elongatus* [23,54,61], it was relevant to know whether GlnB and/or GlnK proteins can perform the same chaperon role in *E. coli*; in which case, the mutations affecting binding to GlnB and/or GlnK and PipX self-interaction should also impair protein levels.

To provide independent evidence of the link between PipX self-interaction and binding to GlnB and/or GlnK proteins, we next analysed the impact of selected *pipX* mutations on PipX levels in *E. coli* BTH101, the host for BACTH assays. To strengthen the analysis with additional mutations while testing the effect of co-expression of the downstream gene *pipY* [31,53] in *E. coli*, we used previously generated shuttle plasmids (Table 1) carrying pipX* (Y6A, Y32A or L65Q in CK1X constructs) and constructed additional ones carrying pipX*pipY (H9A, F12A or F38A in CK1XY constructs). In all cases, a strong constitutive promoter provided by the C.K1 cassette drives the expression of pipX*.

In total, four PipX variants impairing self-interactions (Y6A, H9A, F38A, and L65Q) and two not impairing them (F12A, Y32A) were analysed alongside their corresponding wild-type controls by Western blot analysis with anti-PipX antibodies.

**Figure 7:**
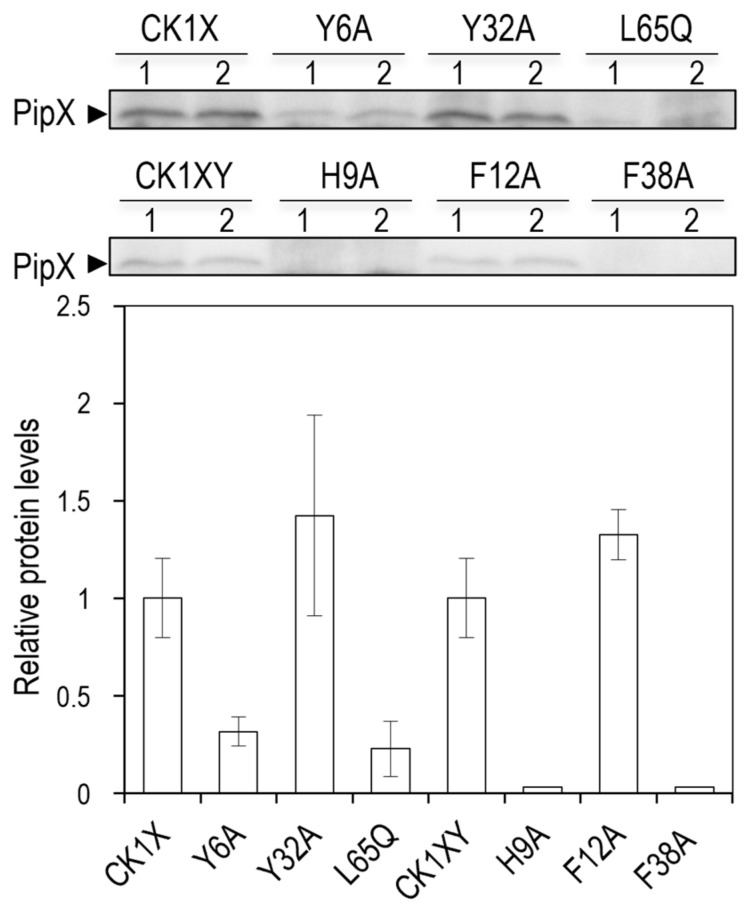
Effects of PipX point mutations (*) on protein levels in *E. coli*. Top: representative immunodetection of PipX*, from CK1X or CK1XY constructions. Bottom: quantification of PipX* band intensities normalised by an unspecific band and referred to WT PipX levels. Data are presented as means and error bars (standard deviation) of four biological replicates.

As shown in Figure 7 (and Figure S3 in the Supplementary Materials), all four variants impairing self-interactions impaired PipX levels, while the other two did not. Importantly, Y32A, which in *S. elongatus* impaired both binding to PII and PipX levels [23], did not decrease PipX levels in *E. coli*. This result, supporting the different effect of mutation Y32A on the interactions with PII and GlnB or GlnK shown in Figure 6, also shows that the interaction with GlnB or GlnK has a positive effect on PipX levels in *E. coli*. Surprisingly, we detected significantly higher levels of PipX from CK1X than from CK1XY constructs (Figure S4 in the Supplementary Materials), a result at odds with that obtained in *S. elongatus*, where PipY had a positive effect on PipX levels [53].

In summary, Western blot analysis confirms the predicted concordance between the effects of PipX mutations on protein levels and on both the self- and cross-interactions of PipX with GlnB and/or GlnK.

### 2.7. Lessons from Two-Hybrid Assays

This study provides, to our knowledge, the first elucidation of the molecular basis of a “false positive” in the BACTH system. We have shown here that PipX-self interaction is not an artefact or the result of massive self-aggregation. Instead, PipX oligomerisation is specifically facilitated by host proteins, a finding that accounts for discordances between bacterial and yeast two-hybrid results discussed in this work. In addition, this study illustrates how proteins that can bind to at least one of the pair of tested proteins can contribute to both “false positives”, that is, indirect interactions, and to false negatives in the BACTH system.

Given the multiplicity of factors that can prevent interactions between pairs of fusion proteins, it is not surprising that false negatives are so frequent in yeast and bacteria two-hybrid systems [62,63,64]. Relevant factors often contributing to false negatives are the absence of appropriated effectors or other components needed for complex formation and the occlusion of interaction determinants within the fusion proteins being studied. Therefore, these factors need to be considered similarly in the context of BACTH and Y2H assays. In contrast, false positives are only frequent in the Y2H system, due to the “stickiness” of the activation domain of GAL4 [65,66]. In the BACTH system, where this type of artefact does not apply, the rare cases of interactions giving positive results appear to inform on the indirect binding facilitated by host proteins, as we have shown here. Since bacterial proteins are less likely to interact with *S. cerevisiae* nuclear proteins than with *E. coli* cytoplasmic or membrane-associated proteins, the Y2H system would be less prone to indirect interactions when testing bacterial proteins.

An important feature of the BACTH system is the interdependency between the strength of interactions and the levels to which protein fusions accumulate, due to the requirement of cAMP for the transcription of fusion genes [56]. Thus, when assaying proteins from phylogenetically distant bacteria, weak interaction signals and false negatives would still be compatible with strong binding affinity between the tested proteins.

A main challenge in the study of signalling networks is to show the physiological context and significance of putative interactions. The BACTH system has been extensively used to demonstrate the specificity of protein interactions [67,68,69,70,71,72]. However, despite initial optimism [41,56,57], reports of using it to approach the regulation of protein complexes are difficult to find [73]. One exception is a very recent work illustrating the importance of cobalamin for BACTH interaction signals between LdaP, the Light-dependent anti-repressor of PspR, (LdaP/PpsR) and PspR [74].

Other strategies to study protein interactions, such as the NanoBiT complementation system based on the reconstitution of the small- and high-output bioluminescence enzyme NanoLuc [75], are far more informative than the BACTH system. Used with both mammalian [76,77,78] and bacterial [79,80,81] cells, the NanoBiT system has recently been used to analyse, as a proof of concept and with unprecedented detail, the regulation of PipX- PII and PipX-NtcA complex formation in *S. elongatus* [61]. However, this remarkably powerful approach, which is yet to be exploited in the study of cyanobacterial interaction networks, requires time-consuming obtention of appropriated *S. elongatus* strain derivatives. In contrast, the comparative simplicity of two-hybrid methods makes them more appropriate for comparative studies of multiple variants of a given protein and to address specific questions on interaction determinants.

## 3. Materials and Methods

### 3.1. Strains, Oligonucleotides and Plasmid Construction

The strains and plasmids used in this work are listed in Table 1, while oligonucleotides are in Table S2 in the Supplementary Materials. Cloning procedures were carried out with *Escherichia coli* XL1-Blue, using standard techniques [82]. The antibiotics used were ampicillin (75 µg mL^−1^), kanamycin (50 µg mL^−1^) or chloramphenicol (17 µg mL^−1^). All constructs were analyzed by automated dideoxy DNA sequencing.

QuickChange mutagenesis was performed using pUAGC934 (to generate for PipX*-T18 derivatives), pUAGC444 (T18-PipX* derivatives) or pUAGC1045 (PipX*-T25 derivatives), as templates. Table S2 summarises the pair primers and template used, with an indication of the resulting plasmids and protein fusions obtained in each case.

*E. coli glnK* sequences were PCR-amplified from *E. coli* MG1655 total genomic DNA with the primers GLNK-BYTH-1F and GLNK-BYTH-1R, digested with *Bam*HI and *Sma*I and cloned into pUT18c, pT25 and pKT25, giving the plasmids pUAG660, pUAG661 and pUAG665, respectively. *E. coli glnB* sequences from pUAG652 were digested with *Bam*HI and *Kpn*I and cloned into pKT25, giving the plasmid pUAG663.

To generate plasmid pUAGC408, *pipXpipY* sequences from pUAGC125 were digested with *Sal*I + *Ksp*AI and cloned into plasmid pUAGC410 cut with *Sal*I + *Sma*I. QuickChange mutagenesis, with pUAGC408 as a template and PipX-H9A-1F/1R, PipX-F12A-1F/1R or PipX-F38A-1F/1R as primers pairs, were used to generate the plasmids pUAGC948, pUAGC939 and pUAGC940.

To generate *E. coli* strains expressing PipX derivatives, BTH101 was transformed with pUAGC410, pUAGC685, pUAGC686, pUAGC682, pUAGC408, pUAGC948, pUAGC939 or pUAGC940.

### 3.2. BACTH Assays

*E. coli* BTH101 was transformed with a pairwise combination of plasmids (50 ng). Five transformant clones from each plate were inoculated into 0.5 mL of LB [83] containing the corresponding antibiotics and 0.5 mM IPTG (Isopropyl β-D-1-thiogalactopyranoside) and incubated at 30 °C for 24 h.

Interactions were assayed by dropping 3 μL of each saturated culture onto M63 ([84]; containing 0.3% maltose, 0.0001% thiamine, 1 mM magnesium sulphate, 0.5 mM IPTG and 40 µg mL^−1^ X-gal) and MacConkey ([85]; containing 1% lactose and 0.5 mM IPTG) reporter plates. Reporter plates were incubated for 24 (MacConkey) or 48 h (M63) at 30 °C and photographs were taken at 24-h intervals.

### 3.3. Protein Extraction and Immunodetection Assays

For the immunodetection assays, cultures were grown in 5 mL LB with Ap (75 µg mL^−1^) overnight at 37 °C, diluted to an OD_600nm_ of 0.05, and grown at 30 °C until they reached an OD_600nm_ of 0.5. Cells were harvested by centrifugation at 7300× *g* for 6 min at 4 °C. The pellets were resuspended in 60 μL of lysis buffer (25 mM Tris/HCl pH 7.5, 0.5 mM EDTA, 1 mM beta-mercaptoethanol and 1 mM PMSF) and the cells were disrupted using a spoon of 0.1 mm glass beads, as described previously [49]. Mixtures were subjected to three cycles of 60 s at a speed of 5 m/s in a high-speed homogeniser, the Minibeadbeater (BioSpec Products, Inc., Bartlesville, OK, USA), always followed by 60 s at 4 °C during each cycle. The samples were centrifuged (5500× *g* for 5 min) and the supernatant fractions (crude protein extracts) were transferred to a new tube and stored at −20 °C until needed.

Protein concentrations were estimated by the Bradford method [86] using the Pierce^TM^ detergent compatible Bradford assay kit (Thermo Fisher Scientific, Waltham, MA, USA) in a VICTOR3^TM^ 1420 multilabel plate reader (PerkinElmer, Waltham, MA, USA). Immunodetection was performed by loading 60 µg of total protein extract in sodium dodecyl sulfate-polyacrylamide gel electrophoresis (Tricine-SDS-PAGE; 18% polyacrylamide). Tricine-SDS-PAGE analysis was conducted according to the method described in [87]. The samples were electrophoresed at a constant voltage of 30 mV until all samples entered the stacking gel and then at a constant voltage of 100 mV until the end of running.

The gel electrophoresis was followed by wet immunoblotting with 0.1 μm polyvinylidene fluoride membranes (from GE Healthcare Technologies, Inc., Chicago, IL, USA). The membranes were blocked with Tris-buffered saline (TBS-tween; 20 mM Tris/HCl pH 7.5, 500 mM NaCl and Tween 20 0.1%) solution containing 5% non-fat dried milk for 30 min at room temperature and then incubated overnight in TBS plus 0.1% Tween 20 solution containing 2% non-fat dried milk and the primary antibody. Then, the membranes were incubated at room temperature for 1.5 h with a 1:150,000 dilution of ECL rabbit IgG, HRP-linked F(ab’)2 fragment (from donkeys; GE Healthcare Technologies, Inc., Chicago, IL, USA). The signal was detected with the addition of the SuperSignal WestFemto reagent (Thermo Fisher Scientific, Waltham, MA, USA) in a Biorad ChemiDoc imager using the automatic exposure mode and avoiding pixel saturation or using X-ray and scanning the films. All the membranes were treated first with a 1:5000 dilution of primary anti-PipX antibody. Anti-serum against PipX (Pineda Antikörper Service, Berlin, Germany) was produced in rabbits.

### 3.4. Computational Methods

Protein intensity levels were quantified from the Western blot images using the ImageJ software, version 1.53 K. Bands were picked up using the “rectangle” function and the area plot corresponding to the intensity was measured with the “wand” tool. Each area from the PipX immunodetection was normalised using the corresponding area of an unspecific inner band and referred to the control strain.

Graphical representations of PipX structure were generated with PyMOL (The PyMOL Molecular Graphics System, Version 1.7.1.7 Schrödinger, LLC, New York, NY, USA).

## 4. Conclusions

In this work, we have used the bacterial two-hybrid system to perform interaction analyses involving PipX-NtcA or PipX-PII complexes from cyanobacteria, as well as heterologous complexes between PipX and GlnB or GlnK, the two PII homologues from *E. coli*. The results obtained here, discussed alongside those from several Y2H studies, provided further insights into the PipX interaction network and allowed us to compare the outcomes between the two most popular genetic systems to study protein–protein interactions.

Interactions that were previously recorded as negative in the yeast system, as well as indirect interactions dependent on host proteins, were detected and explained on the basis of differential features of the Y2H and BACTH systems. To show that PipX self-interaction is due to oligomerisation over GlnB and/or GlnK proteins in *E. coli*, we performed comprehensive mutational analysis, identifying the determinants involved in PipX self- or cross-interactions with GlnB or GlnK and further corroborating the results by Western blot analysis, showing that complex formation with GlnB and/or GlnK from *E. coli* stabilises PipX. The discussions and considerations made here, calling attention to the complexities, advantages and limitations of these two universal biological approaches to studying protein interactions, would help researchers from different fields to make the most of their two-hybrid analyses.

## Figures and Tables

**Table 1:** Strains and plasmids.

Strain	Genotype, Relevant Characteristics	Source or Reference
*E. coli* XL1-Blue	*recA1 endA1 gyrA96 thi-1 hsdR17 supE44 relA1* lac [F′ *proAB* lacI^q^*Z*∆*M15* Tn*10* (Tet^R^)].	[50]
*E. coli* MG1655	F^-^ *ilvG*- *rfb*-50 *rph*-1	[51]
*E. coli* BTH101	F^-^ *cya-99 araD139 galE15 galK16 rpsL1 hsdR2 mcrA1 mcrB1*	[52]
**Plasmid**	**Description, Relevant Characteristics**	**Source or Reference**
pUT18c	CyaA(225–399)T18, Ap^R^	[52]
pT25	CyaA(1-224)T25, Cm^R^	[41]
pKT25	CyaA(1–224)T25, Km^R^	[52]
pUAGC444	T18-PipX, Ap^R^	[21]
pUAGC934	PipX-T18, Ap^R^	[48]
pUAGC1047	T25-PipX, Km^R^	[48]
pUAGC1045	PipX-T25, Km^R^	[48]
pUAGC1048	T25-PII, Km^R^	[48]
pUAGC441	T25-PII, Cm^R^	[21]
pUAGC1075	T25-NtcA, Km^R^	[48]
pUAG652	T18-GlnB, Ap^R^	[21]
pUAG653	T25-GlnB, Cm^R^	[21]
pUAG663	T25-GlnB, Km^R^	This work
pUAG660	T18-GlnK, Ap^R^	This work
pUAG661	T25-GlnK, Cm^R^	This work
pUAG665	T25-GlnK, Km^R^	This work
pUAGC125	pGAD424 with *pipXY* genomic region, Ap^R^	[53]
pUAGC410	[Φ(C.K1-*pipX*)], Ap^R^ Km^R^	[21]
pUAGC682	[Φ(C.K1-pipX^L65Q^)], Ap^R^ Km^R^	[20]
pUAGC685	[Φ(C.K1-*pipX*^Y6A^)], Ap^R^ Km^R^	[23]
pUAGC686	[Φ(C.K1-pipX^Y32A^)], Ap^R^ Km^R^	[23]
pUAGC408	[Φ(C.K1-*pipXpipY*)], Ap^R^ Km^R^	This work
pUAGC939	[Φ(C.K1-pipX^F12A^*pipY*)], Ap^R^ Km^R^	This work
pUAGC940	[Φ(C.K1-pipX^F38A^*pipY*)], Ap^R^ Km^R^	This work
pUAGC948	[Φ(C.K1-pipX^H9A^*pipY*)], Ap^R^ Km^R^	This work

## Data Availability

Data in contained within the article and Supplementary Materials.

## Supplementary Materials

The following supporting information can be downloaded at: https://www.mdpi.com/article/10.3390/ijms25105429/s1.
