# Supplementary material for: Studies on the PII-PipX-NtcA Regulatory Axis of Cyanobacteria Provide Novel Insights into the Advantages and Limitations of Two-Hybrid Systems for Protein Interactions
Source: Int J Mol Sci. 2024 May 16;25(10):5429. doi: 10.3390/ijms25105429 (PMC11121479; doi:10.3390/ijms25105429)
Supplement: Supplementary file 1 [file ijms-25-05429-s001.zip › Tables_suplementary.pdf]

Table S1. Quick-change mutagenesis of PipX derivatives for BACTH analysis.

Primer 1	Primer 2	Template	Resulting plasmid	Fusion protein expressed
pUT18-PipX-E4A-F	pUT18-PipX-E4A-R	pUAGC934	pUAGC1140	PipX^{E4A}-T18
		pUAGC1045	pUAGC1166	PipX^{E4A}-T25
CK1E4A-F	CK1E4A-R	pUAGC444	pUAGC1032	T18-PipX^{E4A}
PipX-Y6A-F	PipX-Y6A-R	pUAGC934	pUAGC1141	PipX^{Y6A}-T18
		pUAGC444	pUAGC1147	T18-PipX^{Y6A}
		pUAGC1045	pUAGC1167	PipX^{Y6A}-T25
PipX-H9A-1F	PipX-H9A-1R	pUAGC934	pUAGC1088	PipX^{H9A}-T18
		pUAGC444	pUAGC800	T18-PipX^{H9A}
		pUAGC1045	pUAGC1168	PipX^{H9A}-T25
PipX-F12A-F	PipX-F12A-R	pUAGC934	pUAGC1089	PipX^{F12A}-T18
		pUAGC444	pUAGC1034	T18-PipX^{F12A}
		pUAGC1045	pUAGC1169	PipX^{F12A}-T25
PipX-Y16A-F	PipX-Y16A-R	pUAGC934	pUAGC1142	PipX^{Y16A}-T18
		pUAGC444	pUAGC1148	T18-PipX^{Y16A}
		pUAGC1045	pUAGC1170	PipX^{Y16A}-T25
PipX-Y32A-1F	PipX-Y32A-1R	pUAGC934	pUAGC1090	PipX^{Y32A}-T18
		pUAGC444	pUAGC801	T18-PipX^{Y32A}
		pUAGC1045	pUAGC1171	PipX^{Y32A}-T25
PipX-R35A-F	PipX-R35A-R	pUAGC934	pUAGC1143	PipX^{R35A}-T18
		pUAGC444	pUAGC1149	T18-PipX^{R35A}
		pUAGC1045	pUAGC1172	PipX^{R35A}-T25
PipX-F38A-F	PipX-F38A-R	pUAGC934	pUAGC1091	PipX^{F38A}-T18
		pUAGC444	pUAGC1086	T18-PipX^{F38A}
		pUAGC1045	pUAGC1173	PipX^{F38A}-T25
PipX-R54C-F	PipX-R54C-R	pUAGC934	pUAGC1144	PipX^{R54C}-T18
		pUAGC444	pUAGC806	T18-PipX^{R54C}
		pUAGC1045	pUAGC1175	PipX^{R54C}-T25
PipX-L65Q-F	PipX-L65Q-R	pUAGC934	pUAGC1145	PipX^{L65Q}-T18
		pUAGC444	pUAGC807	T18-PipX^{L65Q}
		pUAGC1045	pUAGC1176	PipX^{L65Q}-T25
PipX-R70A-F	PipX-R70A-R	pUAGC934	pUAGC1151	PipX^{R70A}-T18
		pUAGC444	pUAGC1152	T18-PipX^{R70A}
		pUAGC1045	pUAGC1177	PipX^{R70A}-T25
PipX-L80Q-F	PipX-L80Q-R	pUAGC934	pUAGC1146	PipX^{L80Q}-T18
		pUAGC444	pUAGC1150	T18-PipX^{L80Q}
		pUAGC1045	pUAGC1178	PipX^{L80Q}-T25

Table S2. Oligonucleotides.

Name	Sequence (5′— 3′)
pT25-seq	5′ TCGGTGACCAGCGGC 3′
pUT18C-seq	5′ GAAACGGTGCCGGCG 3′
pUT18c-sec-R	5′ GGCTTAACTATGCGGC 3′
pUT18-sec-F	5′ TTCACACAGGAAACAGC 3′
pUT18-sec-R	5′ GTCGATGCGTTCGCG 3′
pKT25-sec-R	5′ TGGGTAACGCCAGGG 3′
pKTN25-sec-R	5′ ATGCCAGACTCCCGGTCG 3′
GLNK-BYTH-1F	5′ GAGGGATCCTATGAAGCTGGTG 3′
GLNK-BYTH-1R	5′ TGCCCCGGGTTACAGCGCCGC 3′
pUT18-PipX-E4A-F	5′ TCCCATGGCTTCCGCGAACTACCTCAA 3′
pUT18-PipX-E4A-R	5′ GATGGTTGAGGTAGTTCGCGGAAGCCA 3′
CK1E4A-F	5′ GAGTAATGGCTTCCGCGAACTACCTCAACCATCCC 3′
CK1E4A-R	5′GGGATGGTTGAGGTAGTTCGCGGAAGCCATTACTC 3′
PipX-Y6A-F	5′ CGCTTCCGAGAACGCCCTCAACCATCCCACC 3′
PipX-Y6A-R	5′ GGTGGGATGGTTGAGGGCGTTCTCGGAAGCG 3′
PipX-H9A-1F	5′ CTACCTCAACGCTCCCACCTTCG 3′
PipX-H9A-1R	5′ CGAAGGTGGGAGCGTTGAGGTAG 3′
PipX-F12A-F	5′ CCTCAACCATCCCACCGCCGGATTGCTCTACC 3′
PipX-F12A-R	5′ GGTAGAGCAATCCGGCGGTGGGATGGTTGAGG 3′
PipX-Y16A-F	5′ CCCACCTTCGGATTGCTCGCCCAAATCTGCAGC 3′
PipX-Y16A-R	5′ GCTGCAGATTTGGGCGAGCAATCCGAAGGTGGG 3′
PipX-Y32A-1F	5′ CGCCACTCTTGCTGCTCAGCGCC 3′
PipX-Y32A-1R	5′ GGCGCTGAGCAGCAAGAGTGGCG 3′
PipX-R35A-F	5′ GCCACTCTTTATGCTCAGGCCCTCTTTTTCTCGTAGCC 3′
PipX-R35A-R	5′ GGCTACGAGAAAAAGAGGGCCTGAGCATAAAGAGTGGC 3′
PipX-F38A-F	5′ GCTCAGCGCCTCTTTGCTCTCGTAGCCTTTGATGC 3′
PipX-F38A-R	5′ GCATCAAAGGCTACGAGAGCAAAGAGGCGCTGAGC 3′
PipX-R54C-F	5′ GCTTTGAGCCAATCGGTTGTAATGAAGCGCGGATGTTGG 3′
PipX-R54C-R	5′ CCAACATCCGCGCTTCATTACAACCGATTGGCTCAAAGC 3′
PipX-L65Q-F	5′ GGTCGACAACCGTCAGCGCCAGCTGCGCCGAGATGC 3′
PipX-L65Q-R	5′ GCATCTCGGCGCAGCTGGCGCTGACGGTTGTCGACC 3′
PipX-R70A-F	5′ CCGTCTGCGCCAGCTGCGCGCAGATGCCAGTCTGCAGGAATA 3′
PipX-R70A-R	5′ TATTCCTGCAGACTGGCATCTGCGCGCAGCTGGCGCAGACGG 3′
PipX-L80Q-F	5′ GCAGGAATACAACCAGCAGCAGCAAGTCTTCAAAC 3′
PipX-L80Q-R	5′ GTTTGAAGACTTGCTGCTGCTGGTTGTATTCCTGC 3′
