# Supplementary figures and images for: Studies on the PII-PipX-NtcA Regulatory Axis of Cyanobacteria Provide Novel Insights into the Advantages and Limitations of Two-Hybrid Systems for Protein Interactions
Source: Int J Mol Sci. 2024 May 16;25(10):5429. doi: 10.3390/ijms25105429 (PMC11121479; doi:10.3390/ijms25105429)

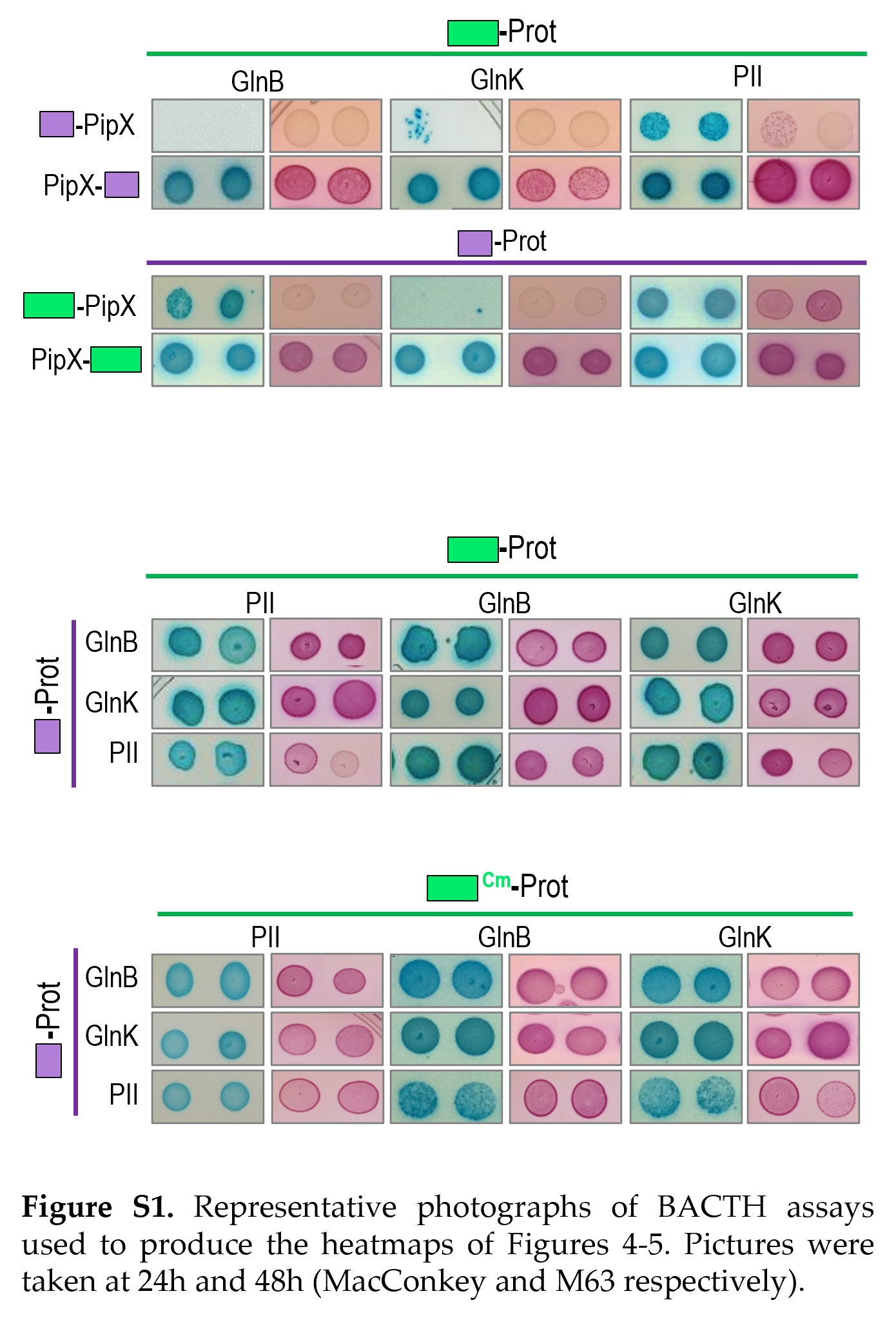

Supplement: Supplementary file 1 [file ijms-25-05429-s001.zip › FigureS1.tif]

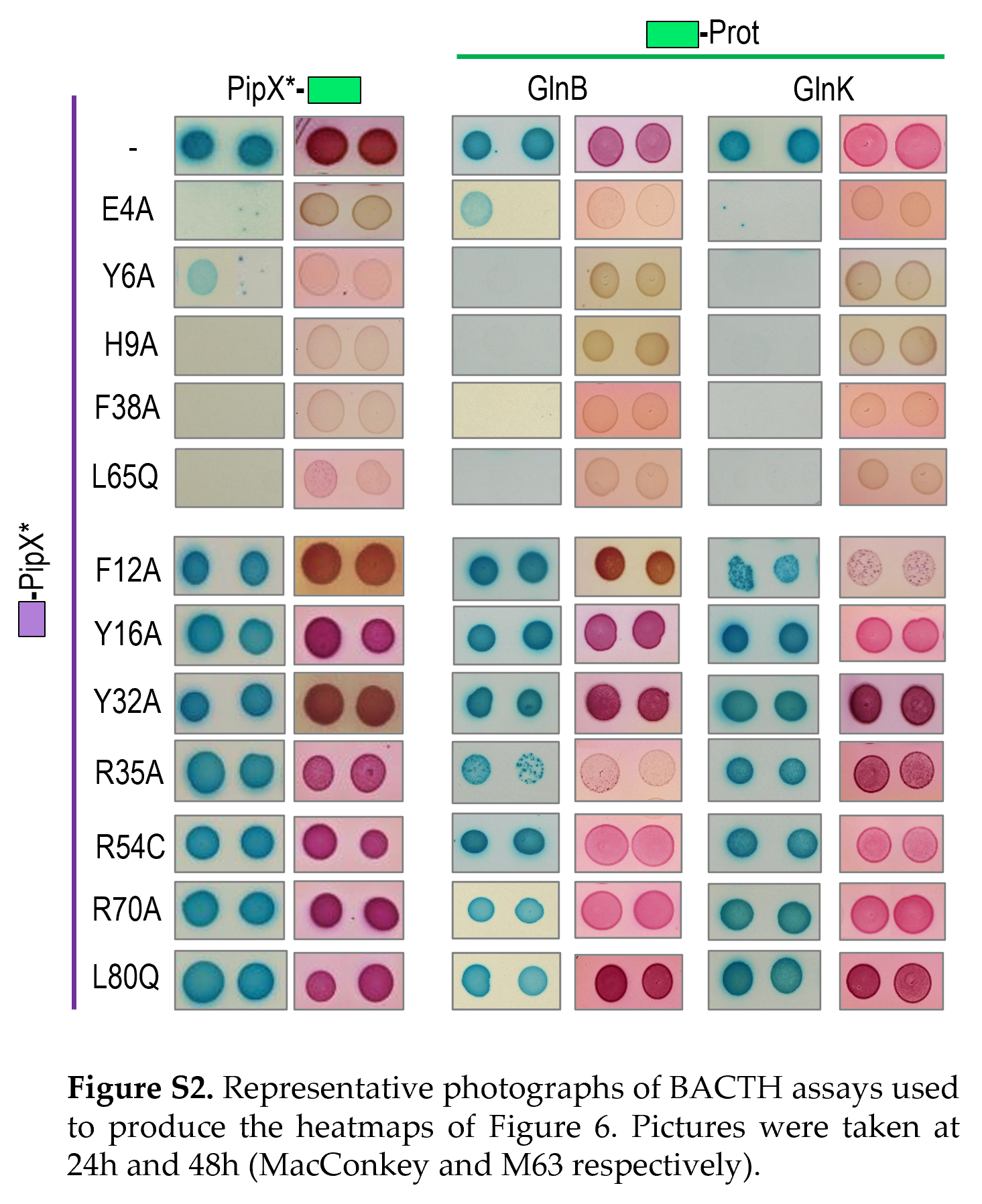

Supplement: Supplementary file 1 [file ijms-25-05429-s001.zip › FigureS2.tif]

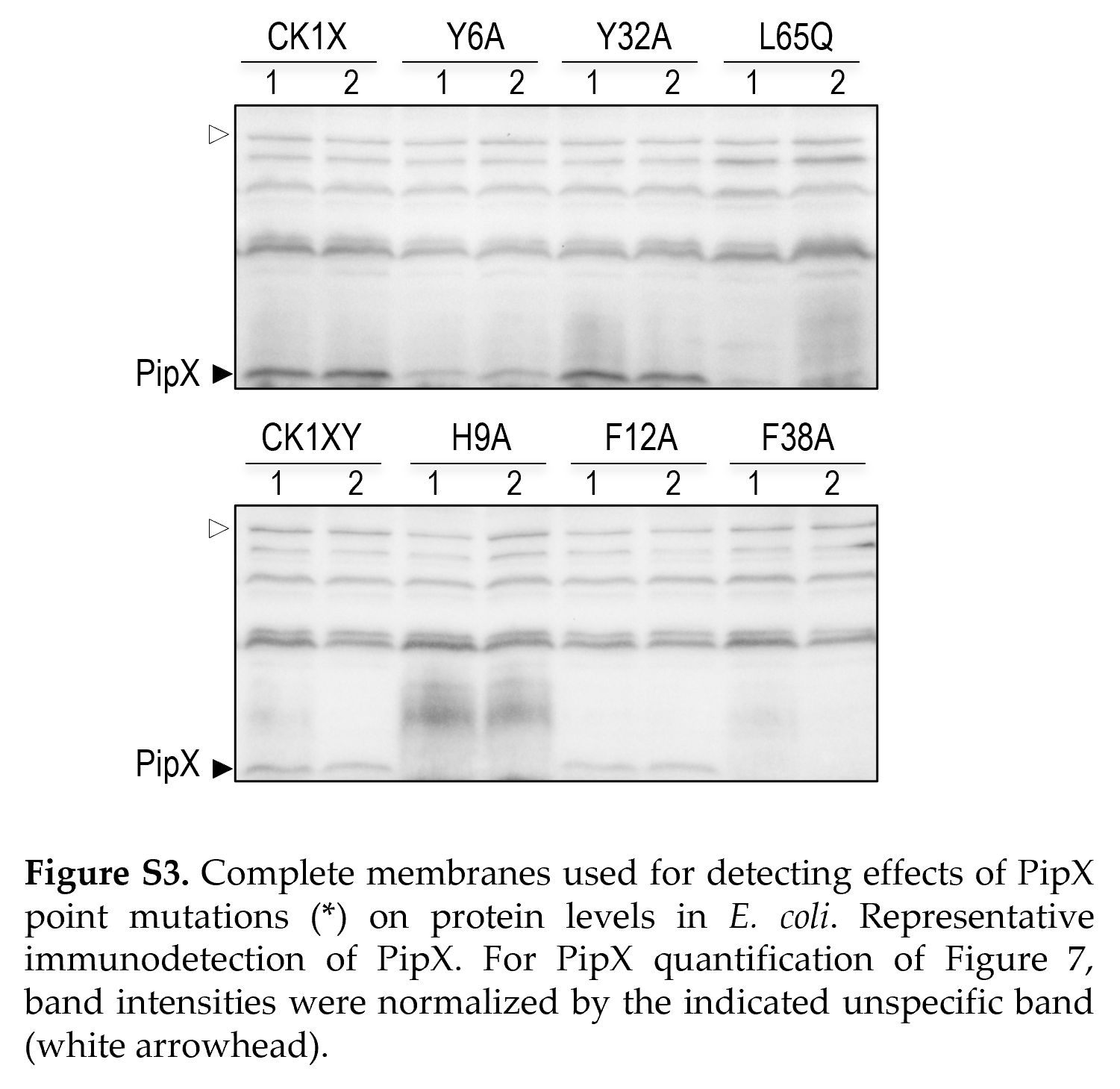

Supplement: Supplementary file 1 [file ijms-25-05429-s001.zip › FigureS3.tif]

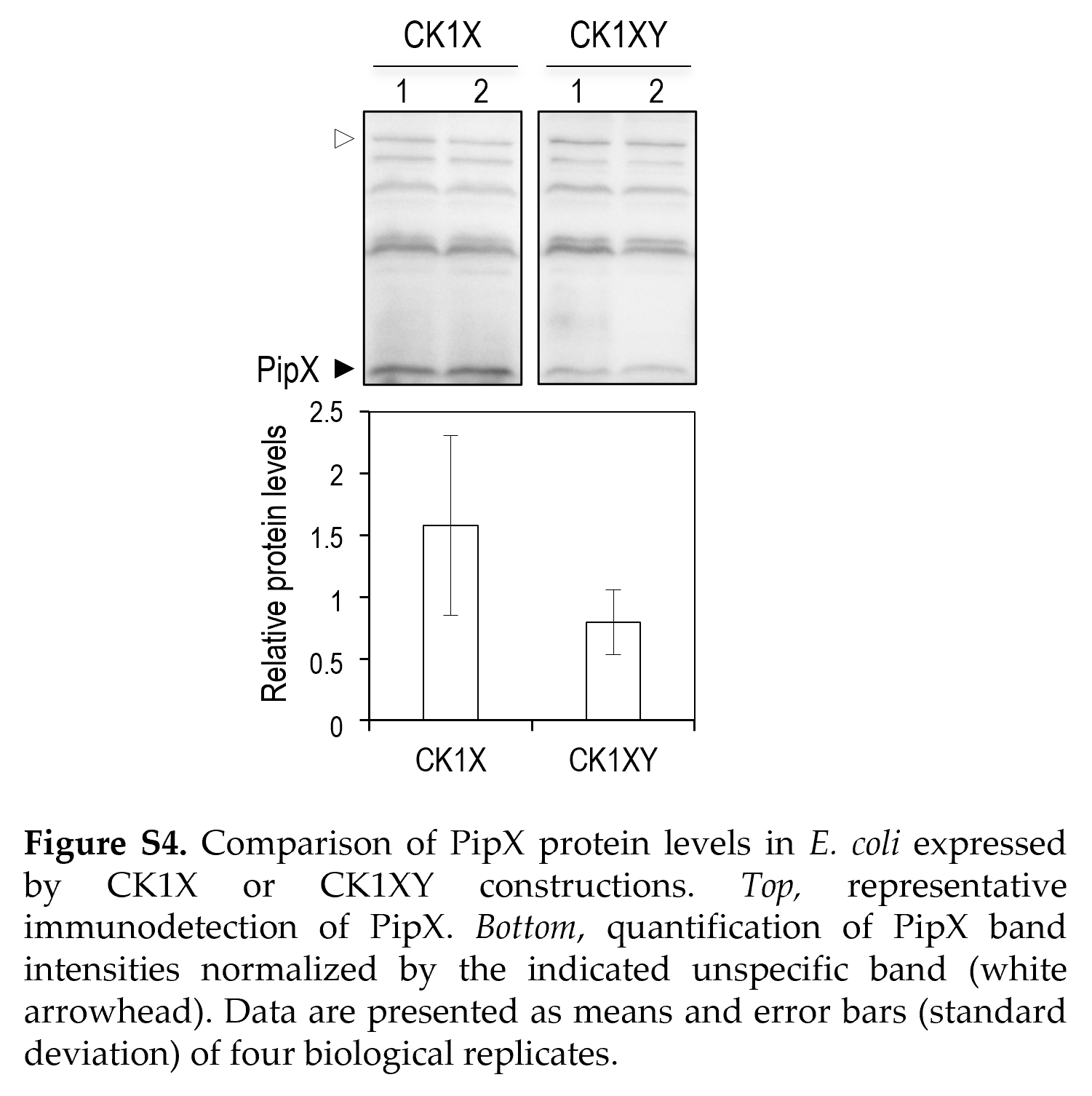

Supplement: Supplementary file 1 [file ijms-25-05429-s001.zip › FigureS4.tif]
